# Compatibility of transvenous implantable cardioverter defibrillator and vagus nerve stimulation device: a case report

**DOI:** 10.1093/ehjcr/ytae214

**Published:** 2024-04-23

**Authors:** Chokanan Thaitirarot, Shirley Sze, Tim Hodson, Ravi Pathmanathan, Harshil Dhutia

**Affiliations:** Glenfield Hospital, University Hospitals of Leicester, Groby Road, Leicester LE3 9QP, UK; Glenfield Hospital, University Hospitals of Leicester, Groby Road, Leicester LE3 9QP, UK; Department of Cardiovascular Sciences, University of Leicester, Leicester, UK; Glenfield Hospital, University Hospitals of Leicester, Groby Road, Leicester LE3 9QP, UK; Glenfield Hospital, University Hospitals of Leicester, Groby Road, Leicester LE3 9QP, UK; Glenfield Hospital, University Hospitals of Leicester, Groby Road, Leicester LE3 9QP, UK

**Keywords:** Implantable cardioverter defibrillator, Vagus nerve stimulation, Device interaction, Epilepsy, Case report

## Abstract

**Background:**

Vagus nerve stimulation (VNS) is an established therapy for drug-resistant epilepsy and depression. While VNS co-existence with cardiac pacemakers is considered safe, its interaction with implantable cardioverter defibrillators (ICDs) remains poorly understood. The concern revolves around the potential for VNS stimulation to interfere with ICD function, potentially resulting in inappropriate therapy or changes in cardiac pacing.

**Case summary:**

We present the case of a 50-year-old woman with drug-resistant epilepsy who underwent VNS device implantation and subsequent transvenous ICD placement for primary prevention post-myocardial infarction. These devices were thoughtfully situated contralaterally, with a minimum 10 cm separation. Comprehensive testing and follow-up demonstrated no interactions during device programming or serial assessments. Simultaneous interrogation of both devices with their respective telemetry wands caused chaotic artefacts in all channels on the ICD, likely due to electromagnetic interference. Importantly, this interference did not affect ICD sensing.

**Discussion:**

The co-existence of VNS and ICD in a patient is an emerging scenario with limited previous reports, yet our findings align with prior cases involving VNS and pacemakers. Emphasizing the need for optimal device separation and meticulous evaluation, particularly at maximum VNS output and ICD sensitivity settings, ensures their safe and feasible co-existence. As the use of VNS alongside cardiac implantable electronic devices becomes more common, a diligent evaluation for potential interactions is imperative. Our case highlights the successful co-existence of VNS and ICD, underscoring the importance of careful monitoring and evaluation to guarantee the safe utilization of these two devices.

**Learning points**

- Concurrent use of VNS and transvenous ICD is feasible and safe.
- Maximizing the distance between the two devices should minimize risk of device interaction and damage to VNS in the event of ICD therapy.
- Close and careful evaluation for interaction is performed at maximum output of VNS and highest sensitivity of ICD must be performed to exclude any interaction.

## Introduction

Vagus nerve stimulation (VNS) is an effective therapy for drug-resistant epilepsy and depression in adults,^1,2^ and can improve symptoms in patients with heart failure (HF).^3,4^ The therapy involves delivering electrical pulses to the cervical portion of the vagus nerve from a device that is commonly implanted in the left infraclavicular area. It is generally safe and well-tolerated, although rare cases of severe sinus bradycardia, atrioventricular block, and ventricular standstill from parasympathetic overstimulation have been reported.^5–7^ While simultaneous use of a VNS device and a cardiac pacemaker has been reported to be safe,^8–10^ the long-term safety of concomitant use of a VNS device and an implantable cardioverter defibrillator (ICD) is not clear. The main concern is potential noise artefacts from the VNS leading to abnormal sensing by the ICD, resulting in inappropriate therapy. Here, we present a rare case of a patient with a VNS device who subsequently underwent transvenous ICD implantation for primary prevention, with a focus on studying possible interference between the devices.

## Summary figure

**Figure:**
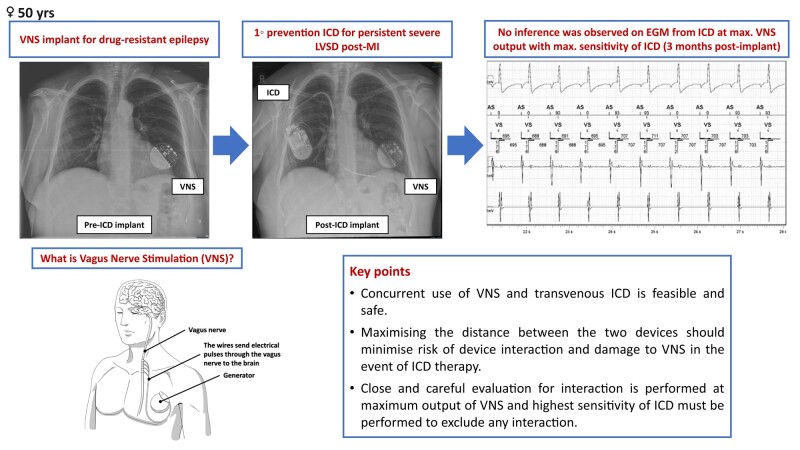


## Case report

A 50-year-old woman suffering from drug-resistant epilepsy was implanted with a left-sided VNS device (Model 105 AspireHC, LivaNova Inc.) in the left infraclavicular region. In the years following the implant, the frequency and severity of seizures had significantly reduced although she still experienced two episodes of short-lasting seizures per week.

She was admitted following an anterior ST elevation myocardial infarction. Emergency coronary angiogram showed acute occlusion at the proximal left anterior descending coronary artery, and she received primary percutaneous coronary intervention. A transthoracic echocardiogram (TTE) demonstrated severe left ventricular (LV) systolic dysfunction with an LV ejection fraction (LVEF) of 30%. She received guideline-directed medical therapy for heart failure with reduced ejection fraction (HFrEF), including bisoprolol 5 mg o.d., sacubitril/valsartan 24/26 mg 1 tablet b.i.d., dapagliflozin 10 mg o.d., and eplerenone 25 mg o.d.

**Figure 1:**
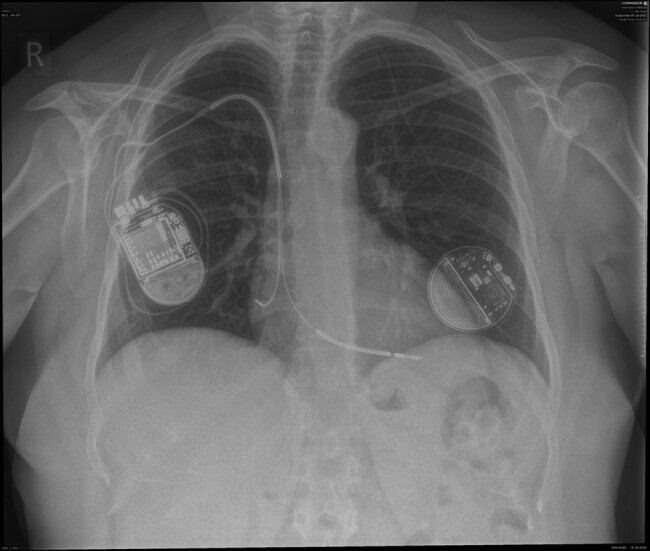
Chest X-ray shows a right-sided transvenous dual-chamber implantable cardioverter defibrillator (ICD) and a left-sided vagus nerve stimulation (VNS) device.

Three months later, a repeat TTE demonstrated persistent LV impairment (LVEF 32%). She has NYHA class II symptoms with a NT-proBNP level of 627 pg/mL. A 12-lead ECG demonstrated sinus rhythm with a heart rate of 60 b.p.m. and a QRS duration 102 ms. A primary prevention ICD was offered following the European Society of Cardiology (ESC) guidelines.^11^ A right-sided transvenous dual-chamber ICD was successfully implanted. Fifty-eight centimetre dual-coil DF4 and 46 cm IS1 MRI-compatible active fixation leads (Durata and Tendril, St. Jude Medical) were positioned in the right ventricular (RV) apex and right atrial appendage, respectively. These leads were then connected to an MRI-compatible generator box (Fortify Assura DR, St. Jude Medical) (*Figure 1* ).

At the end of the procedure, continuous ECG and electrogram (EGM) monitoring was performed to assess for possible interference between the VNS and ICD. Right ventricular sensing was set using true bipolar sensing. No interference was observed in both true bipolar (i.e. tip to ring) and unipolar (i.e. coil to can) RV channels as the output of the VNS was gradually increased to 2.5 mA (the patient developed frequent coughing at this point) at the maximum sensitivity setting of the ICD (i.e. 0.3 mV). Prior to discharge, the device was programmed DDI (40–110 b.p.m.) with the sensitivity of the true bipolar RV sensing set to ‘Auto’.

**Figure 2:**
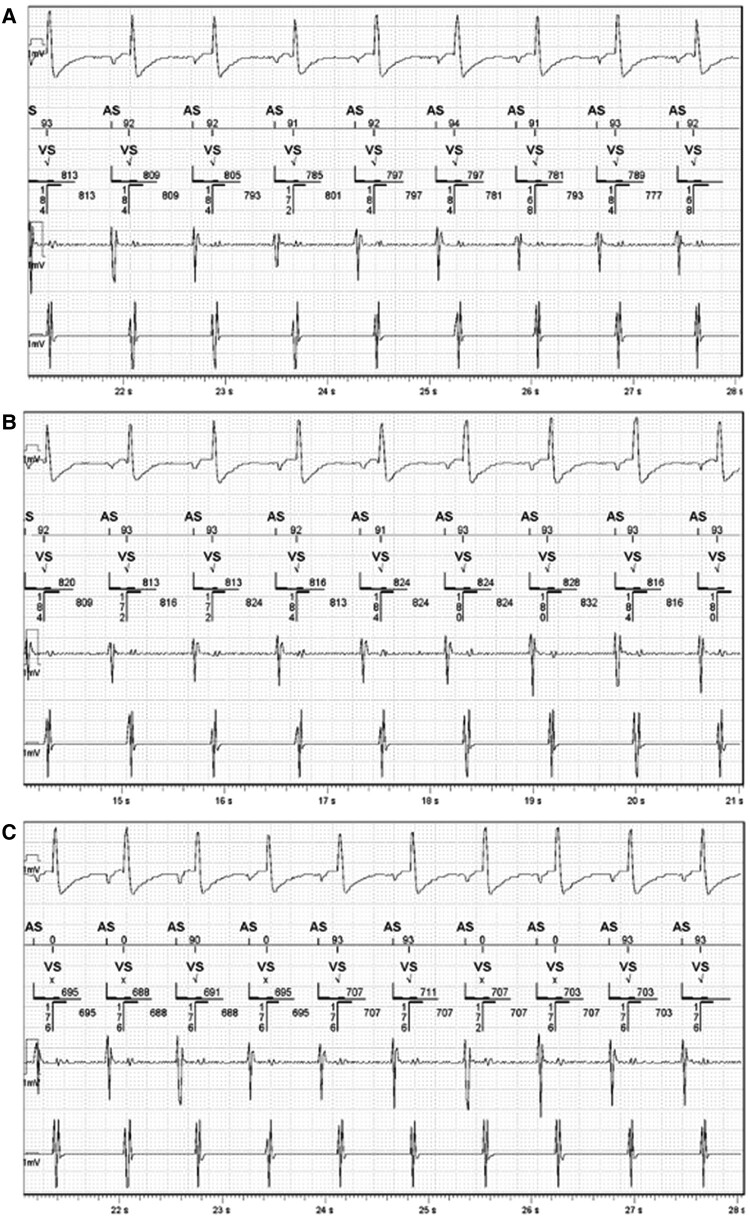
Electrograms (EGMs) from the implantable cardioverter defibrillator (ICD) at different outputs from the vagus nerve stimulation (VNS) device. From top to bottom: coil to can channel, marker channel, true bipolar RA channel, true bipolar RV channel. No interference was observed in all the channels at different VNS outputs (*A*) 0 mA, (*B*) 1.75 mA (patient’s usual setting), (*C*) 2.5 mA (the patient developed frequent coughing) with the sensitivity of the true bipolar RV sensing set to 0.3 mV.

**Figure 3:**
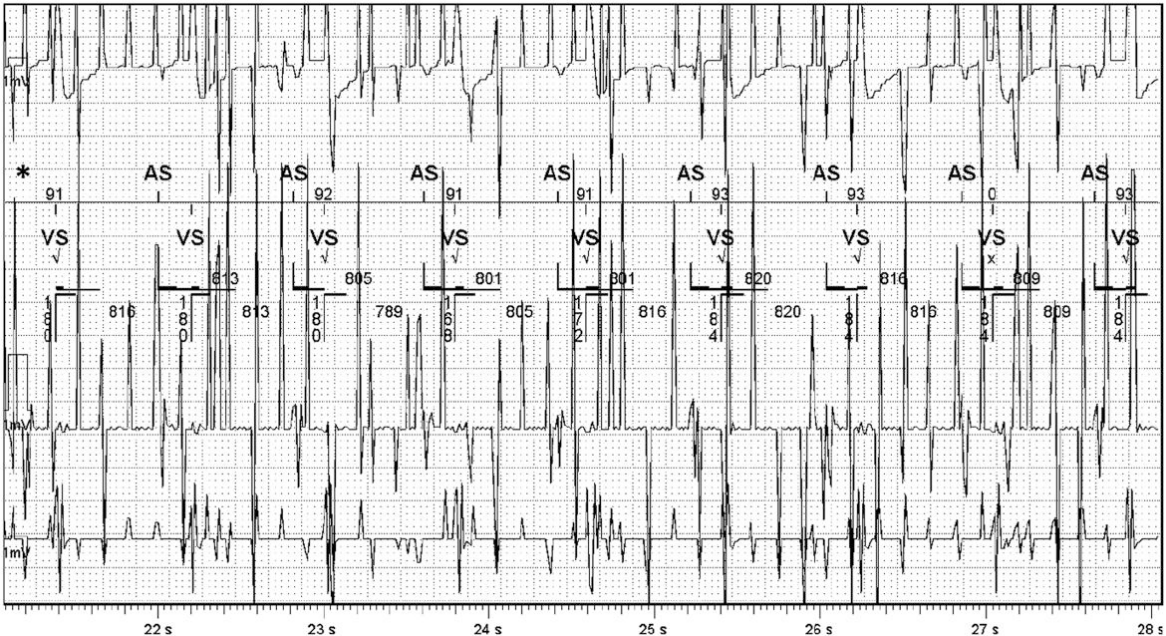
Electrogram from the implantable cardioverter defibrillator (ICD) when the ICD and vagus nerve stimulation (VNS) device were simultaneously interrogated with their respective telemetry wands. There were chaotic artefacts appearing in all the channels with appropriate sensing from the ICD. These are likely to be due to electromagnetic interference from the telemetry wands. One As marker (*) did not appear on the marker channel, possibly because of transient loss of telemetry. During this period, it was impossible to interrogate the VNS device.

The patient was reviewed 3 months post-ICD implantation. She reported no symptoms, and the ICD site was adequately healed. Interrogation of the ICD was unremarkable. No therapy was delivered, nor were there any alerts of concern. Both atrial-pacing (Ap) and ventricular-pacing (Vp) burdens were <1%. Likewise, interrogation of the VNS did not reveal any abnormalities. The frequency of seizures was unchanged. A magnet was used occasionally to activate on-demand VNS in order to prevent or shorten a seizure. Testing was repeated to assess for interference between the devices. *Figure 2* shows EGMs (set as true bipolar RV sensing) from the ICD at different outputs from the VNS. Again, no interference was observed, even during the on-demand VNS activation by a magnet. However, when both devices were interrogated at the same time using their respective telemetry wands, chaotic artefacts appeared in all the channels, although this did not affect sensing from the ICD (*Figure 3*). The artefacts were likely due to electromagnetic interference from the telemetry wands. One atrial-sensed (As) marker did not appear on the marker channel, possibly because of transient loss of telemetry. During this period, it was not possible to interrogate the VNS device.

## Discussion

To our knowledge, this is the first comprehensive description of an adult patient with co-existing VNS and transvenous ICD systems. Partial remission of the epilepsy was successfully achieved in our patient using the VNS device. Interactions with the ICD did not occur during device interrogation, programming, or serial testing over the observation period.

Potential interaction of VNS and cardiac implantable electronic devices (CIEDs), such as ICDs and pacemakers, is poorly understood. The main concern is that stimulation from VNS devices could be detected by CIEDs, leading to delivery of inappropriate therapy and/or a change in cardiac pacing. Concurrent VNS and pacemaker implantation appears to be feasible and safe.^8–10^ However, the co-existence of VNS and ICD is rarely reported. Muller *et al*.^12^ described a case of a 56-year-old male with bipolar disorder and ‘syncope and ventricular tachyarrhythmias’ who had a VNS device and an implanted ICD. No interaction was reported between the devices. Pelargonio *et al*.^13^ reported a case of a 7-year-old girl with long QT syndrome, hypertrophic cardiomyopathy, and drug refractory epilepsy. She had an ICD implanted via epicardial approach followed by ipsilateral VNS implantation. Transient sub-threshold noise in the far-field recording (i.e. can to coil) was detected on the ICD EGM during VNS activation and optimization without compromising the functioning of the ICD. These findings are largely in line with our observations in this case.

In our patient, we implanted the transvenous ICD contralateral to the VNS system. This approach has several advantages over alternative options such as ipsilateral transvenous ICD and subcutaneous ICD (S-ICD). Firstly, it maximizes the distance between the two devices (at least 10 cm as per manufacturer’s recommendation), thereby minimizing the risk of device interaction. Secondly, it minimizes risk of damage to the VNS in the event of ICD therapy. There has been a recent report of inappropriate shocks from a wearable cardiac defibrillator in proximity to the VNS, resulting in malfunctioning of the VNS, leading to symptomatic bradycardia and ventricular arrhythmia.^14^

As a shared decision with the patient, we did not perform defibrillation threshold testing (DFT) at implant given the primary prevention indication, excellent RV sensing, and use of a dual-coil lead and a high energy generator. This is consistent with practice in the UK where DFT is performed routinely in only 27.6% of right-sided ICD implants.^15^ A S-ICD was felt not to be appropriate in this case given concerns of potential electrical interference due to proximity to the VNS, and resting bradycardia (55–60 b.p.m.) that may preclude optimization of beta-blocker therapy for HFrEF.

## Conclusion

Vagus nerve stimulation has proved to be an effective treatment for drug refractory epilepsy and depression, and can improve symptoms from HF. We anticipate concurrent use of a neuromodulator, such as VNS, and a CIED, such as ICD or pacemaker, will be increasingly common in the near future. It is crucial that close and careful evaluation for interaction is performed at the maximum output of the VNS and the maximum sensitivity of a pacemaker or an ICD. We have demonstrated that co-existence of VNS and ICD in the same patient is safe and feasible.

## Data Availability

Data are available upon reasonable request from the author at chokanan.thaitirarot@nhs.net.

## Lead author biography

Dr Chokanan Thaitirarot is a consultant cardiologist with subspeciality in heart failure and device therapy in the UK and Thailand. He is a CCDS-certified cardiac device specialist. He also possesses an MSc in Evidence-Based Health Care from the University of Oxford. Dr Thaitirarot’s research includes a systematic review on risk prediction models for incident heart failure in the community. He has actively engaged in teaching, quality improvement, and various medical societies.

**Consent:** The authors confirm that written consent for submission and publication of the clinical case including image(s) and associated text has been obtained from the patient in line with COPE guidance.

**Funding:** None declared.
